# On External Applications

**Published:** 1898-06

**Authors:** 


### On External Applications.

Friedlaender (*Der Kinderarszt*, 1897, *lxxxvii.*, 63) devotes a short discussion to the rules for external application, from which the following practical hints may be abstracted: On the application of cold, an active contraction of the blood vessels is produced not only in the skin, but to a certain extent in the deeper-lying tissues. A collateral hyperemia occurs in those portions which are not affected by the cold. As long as the cold acts continuously, the contraction of the blood vessels will continue, and this of necessity is bound to produce a slowing of the circulation, a defective flow of blood to these parts, and consequently a retardation of cellular activity and a slowing of the physiological and pathological processes of metabolism. In addition, cold acts on the sensitive nerves in a resolvent manner.

When warm compresses are used, a passive dilatation of the blood vessels accompanied by lowering of tone, slowing of the circulation, and increased leucocytosis from paresis of the vaso-constrictors results. By the use of stimulating or Priesnitz compresses, an active dilatation of the blood vessels, accompanied by retention of the tone, acceleration of the circulation, and increase of the red-blood corpuscles, is caused through excitation of the vasso-dilators.—*Pediatrics.*

